# 

**Published:** 1907-12

**Authors:** 


### The American Journal of Dental Science

The Official Organ of the Florida, Georgia and South Carolina State Dental Societies

Third Series

VOL. XXXVIII DECEMBER, 1907 NO. 12

EDITORS

WM. GIRD BEECROFT, D. D. S., Madison, Wis. B. H. TEAGUE, D. D. S., Aiken, S. C.

Published on the 10th day of each month.
Subscription price $1.00 per year.
ENTERED AS SECOND-CLASS MATTER, MADISON, WIS.

Address all communications, both business and editorial, to
The Dental Publishing Company, Madison, Wisconsin

